# Study on the new strategy and key techniques for accurate prevention and treatment of nonalcoholic steatohepatitis based on intestinal target bacteria

**DOI:** 10.1097/MD.0000000000022867

**Published:** 2020-12-11

**Authors:** Lili Zhuo, Jiali Xu, Ningning You, Liyan Wang, Yu Song, Yan Luo, Junping Shi

**Affiliations:** a Department of Endocrinology, Hangzhou Normal University Affiliated Hospital; b Department of Liver Diseases, Hangzhou Normal University Affiliated Hospital; c Departments of Gastroenterology, Taizhou Hospital of Zhejiang Province Affiliated to Wenzhou Medical University, Taizhou, Zhejiang, China; d Department of Liver Diseases, Zhejiang Chinese Medical University; e Institute of Translational Medicine, Hangzhou Normal University Affiliated Hospital, Hangzhou, Zhejiang, China.

**Keywords:** clinical trial, gut microbiota, nonalcoholic fatty liver disease, soluble dietary fiber

## Abstract

**Background:**

Nonalcoholic fatty liver disease (NAFLD) has emerged as a major health problem worldwide; according to statistics, 10% to 25% of patients with NAFLD can progress to nonalcoholic steatohepatitis (NASH). A link between the composition and metabolites of intestinal microbiota and the development of NAFLD is becoming clearer. It is believed that microbiota factors are driving forces of hepatic steatosis and inflammation. The formulated food that contains prebiotics and dietary fiber may improve NAFLD by altering the intestinal flora and its metabolites.

**Methods:**

The study plan to recruit adult patients (18–75 years, n = 120) with NAFLD, range of alanine aminotransferase is 1.5 to 5 times upper limit of normal (ULN) or liver biopsy is confirmed as NASH. Participants will be randomly allocated into 2 groups: formulated food (n = 80) and a placebo group (n = 40) for 24 weeks. Both groups will receive lifestyle and nutritional advice. The primary endpoint is a decrease in MRS-PDFF by more than 30% from baseline at 24 weeks. The secondary endpoints include the change of anthropometric, liver function, glycolipid metabolism, and systemic inflammation at 4, 12, and 24 weeks. In addition, we consider the changes in intestinal microbiota as an exploration to assess the abundance and diversity at 24 weeks. Weeks 24 to 36 are the follow-up period of drug withdrawal.

**Discussion:**

This clinical trial will provide evidence of efficacy and safety of formulated food as a potential new therapeutic agent for NAFLD patients.

**Trial Registration:**

The trial is registered in the China Clinical Trial Center (ChiCTR1800016178).

## 1 Introduction

Nonalcoholic fatty liver disease (NAFLD) is a chronic progressive liver disease caused by accumulation of intrahepatic fat. With the significant increase in the incidence of obesity and type 2 diabetes, NAFLD has surpassed chronic hepatitis B to become the most common chronic liver disease in China. According to statistics, 10% to 25% of patients with NAFLD can progress to nonalcoholic steatohepatitis (NASH), of which about 20% progress to cirrhosis, and liver cancer have been increasing rapidly in recent years.^[1]^

The current treatment of NAFLD mainly includes Lifestyle intervention: refers to weight control through reasonable diet and exercise. There is evidence that the improvement of liver histology is related to weight loss. When weight loss is 10%, liver histology (steatosis, inflammation, and fibrosis) can be relieved, but due to poor compliance, only 9.9% of patients lose weight within 1 year more than 10%.^[2,3]^ In addition, whether exercise has independent benefits for NAFLD is still inconclusive. Drug therapy: Some drugs such as insulin sensitizers and antioxidants can improve the effect, but they are not approved for the treatment of NAFLD. These drugs have their limitations in treatment. For example, pioglitazone can improve liver steatosis, insulin resistance, and liver enzyme levels, but weight gain of about 3 to 5 kg is the most common adverse reaction,^[4]^ and patients with obvious heart failure also It should not be taken, and it is also related to bladder cancer and bone loss after menopause.^[5–8]^ Human glucagon-like peptide-1 (GLP-1) analogs, such as liraglutide, can suppress appetite, delay gastric emptying, and reduce weight. However, GLP-1 receptors may be downregulated after long-term activation, and the drug may be stopped. Debounce may occur afterwards. In addition, PIVENS studies have shown that antioxidant vitamin E can significantly improve liver inflammation in nondiabetic-NASH patients, but it is not effective for liver fibrosis and is related to the risk of prostate cancer and hemorrhagic stroke,^[9–12]^ so safety needs further evaluation. Surgical treatment: Bariatric surgery can significantly improve inflammation and ballooning, but it is an invasive operation and is not recommended. Therefore, the actual treatment of NAFLD has not been resolved.

Current studies have shown that the occurrence and development of NAFLD are closely related to the composition and metabolites of the intestinal flora.^[13]^ In obese patients, the diversity and abundance of intestinal microbes have undergone significant changes, prone to overgrowth of small intestinal bacteria, increased intestinal permeability, and increased absorption of endotoxins,^[14,15]^ thereby activating the NF-kB pathway and its related inflammatory pathways increase the possibility of NAFLD formation.^[16,17]^

It has been projected that dietary factors play a more important role in shaping the composition of the gut microbiota.^[18]^ At present, it is mainly regulated by probiotics, prebiotics, and synbiotics. Prebiotics are defined as “a selective fermentation component that causes a specific change in the composition and/or activity of the gastrointestinal microbiota to confer a host's health benefit.”^[19]^ In pig microbiome analysis, high-fiber/low-fat diet is associated with higher concentration of *Bifidobacteria*, *Lactobacilli*, and *Faecalibacterium prausnitzii*, which have a protective role in intestinal inflammation.^[20]^ This phenomenon also exists in a human experimental research that healthy subjects with improved glucose metabolism following fiber supplementation have a higher *Prevotella/Bacteroides* ratio than those who do not respond to increased fiber.^[21]^

Fermentation of fiber by gut microbiota yields SCFAs that not only provide energy for the host but also play an immune regulatory role. Recent studies have shown that SCFAs exerts anti-inflammatory and immune effects through the effects on Treg cell expansion/generation via SCFAs-GPCR or their HDAC-inhibiting ability.^[22]^ Prebiotics can also improve intestinal barrier function and alleviate inflammation and insulin resistance associated with obesity by increasing gut hormone release, such as glucagon-like peptides 1 and 2 (GLP-1 and GLP-2), and by regulating endogenous cannabinoid systems.^[13,23,24]^ In addition, Bomhof et al^[25]^ through liver biopsy follow-up found that the overall NAS score decreased after taking prebiotics.

These results suggest that improving the gut microbiota by taking dietary supplements may be a potential treatment for patients with NAFLD. The most commonly used prebiotics in practice are oligofructose, galacto-oligosaccharide, lactulose and nondigestible carbohydrate inulin, cellulose, resistant starch, hemicellulose, gums, and pectin, while formulated food is rich in prebiotics, mainly oligofructose, isomalto-oligosaccharide, and dextrin. Therefore, we designed a randomized, double-blind, placebo-controlled trial to evaluate the role of formulated food in improving NAFLD.

## 2 Methods

### 2.1 Trial design

The aim of the trial is to evaluate the efficacy and safety of formulated food 2 packs per day vs placebo for 24 weeks in NAFLD. During this time, subjects need to visit the hospital regularly and comply with an individualized diet and lifestyle provided by a professional dietitian, at the same time increasing exercise, the frequency is maintained 4 to 5 times a week, 40 minutes each time. Dietary intake at these stages will be recorded by an app for easy management assessment. The study plans to recruit 120 adult NAFLD patients from the Affiliated Hospital of Hangzhou Normal University, randomly assigned.

### 2.2 Ethical considerations and registration

The study protocol is in line with the Helsinki Declaration and related laws. We conducted an ethical review in accordance with this declaration and regulations, and the trial was approved by the Ethics Committee of the Hangzhou Normal University Affiliated Hospital. The trial was also registered with the China Clinical Trial Center with registration number ChiCTR1800016178. All subjects will sign a written informed consent form and retain a copy before receiving treatment, which have been approved by the qualified Ethics Committee. During and after the study, we will follow the requirements of the Ethics Committee.

### 2.3 Eligibility

In the NAFLD patients enrolled in this study, we require ALT>1.5ULN or liver biopsy to confirm NASH with a 25 kg/m^2^< BMI <35 kg/m^2^. In addition, we excluded patients who had been taking drugs, which can influence ALT in the past 3 months. Detailed enrollment and exclusion criteria are summarized in Tables 1 and 2.

**Table 1:** Inclusion criteria.

Criteria type	Description of inclusion criteria
Sex	Men and women
Age	18–75 yr
Alcohol consumption	No history of significant alcohol consumption (<70 g/wk for women and < 140 g/wk for men)
Body mass index	25 kg/m^2^<BMI<35 kg/m^2^
Evidence of NAFLD	ALT>1.5×ULN at the start of this study, or liver biopsy to confirm NAFLD
Blood pressure	Hypertension patients will be required to take a stable antihypertensive drug(s) to keep blood pressure stable (<140/90 mm Hg) 2 mo before randomization, patients may continue to take antihypertensive drug(s) during clinical trials
Blood lipid	If a participant is using a statin or fibrate, he/she will be required to be on a fixed dose to keep lipids stable within 3 mo before enrollment
Other drug use	The following medications were not received within 3 mo before enrollment (metformin, thiazolidinediones, hypoglycemic agents, DPP-4 inhibitors, GLP-1, SGLT2 inhibitors, polyene phosphatidylcholine, glycyrrhizin preparation, bicyclol, reduced glutathione, S-adenosylmethionine, silymarin, OCA/UDCA, betaine, fish oil, phosphodiesterase inhibitor, gemfibrozil)
Informed consent	Understand the study and sign an informed consent form

BMI = body mass index, ALT = alanine aminotransferase, DPP-4 = dipeptidyl peptidase-4, GLP-1 = glucagon-like peptide-1, SGLT2 = sodium-dependent glucose transporters 2, OCA = obeticholic acid, UDCA = ursodeoxycholic acid.

**Table 2:** Exclusion criteria.

Criteria type	Description of exclusion criteria
Alcohol consumption	Alcohol and/or drugs abuse and dependence over the past 5 yr History of significant alcohol consumption (>10 g/day for women and >20 g/day for men)
Liver comorbidities	Patients with cirrhosis Patients with any other acute or chronic active liver diseases such as viral hepatitis, hereditary hemochromatosis, hepatolenticular degeneration, alpha-antitrypsin deficiency, alcoholic liver disease, drug-induced liver diseases, etc
Other comorbidities	Known heart failure of New York Heart Association class 2, 3, or 4 Known acceptance of cardiac pacemaker implantation Uncontrollable hypertension With uncontrolled hypothyroidism (serum level of thyroid-stimulating hormone is 2 times higher than the upper limit of normal value) Renal insufficiency, serum creatinine > upper limit of normal Evidence of positive human immunodeficiency virus antigen or antibody (HIV-Ag/Ab)
Other	Pregnant or lactating women Female early pregnancy screening test positive, or unwilling to contraception during clinical trials Cannot accept MRI examiners Other investigators believe that clinical trials cannot be completed Those who cannot sign informed consent

### 2.4 Study flow and schedule

**Figure 1:**
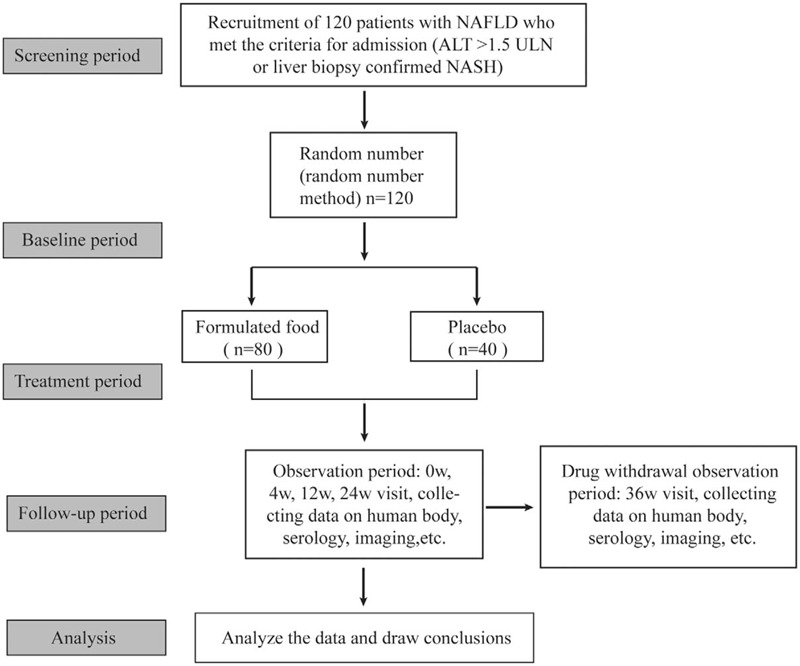
Study design.

The flow chart of the study is shown in Figure 1, and the study schedule is summarized in Table 3. For study visits at week 0, week 4, week 12, week 24, week 36 patients must fast for 8 hours before the visit, and then take blood for metabolic assessment. Body weight, waist circumference, and hip circumference will be measured at each visit. In order to better assess patient compliance, we require that remaining meal powders be returned at each study visit and will be counted by drug administrators to further reduce the likelihood of unblinding.

**Table 3:** Objectives and procedures.

	Study time point, wk				
	Treatment period				Follow-up period
Study objectives	0	4	12	24	36
Primary objectives					
Change in liver fat content (MRS/MRI-PDFF)	×		×	×	
Secondary objectives					
Change in anthropological indicators (BMI and WHR)	×	×	×	×	×
Change in liver function (ALT, AST, GGT)	×	×	×	×	×
Change in diabetic factors (FPG, FINS, HOMA-IR, HbA1c)	×	×	×	×	×
Change in lipids (TG, TC, HDL-C, LDL-C)	×	×	×	×	×
Change in IL-6 and CK18	×	×	×	×	×
Change in HRQOL (SF-36)	×			×	
Assessment of diet and exercise	×	×	×	×	
Record distribute and recycle of drug	×	×	×	×	
Record of drug combination	×	×	×	×	
Safety objectives					
Renal function (BUN, Cr, eGFR)	×	×	×	×	×
Blood routine	×	×	×	×	×
Urine routine	×	×	×	×	×
Fecal routine	×				
Coagulation function	×				
TSH	×				
Virus	×				
Blood HCG	×				
ECG	×			×	
Exploratory endpoint					
Change in intestinal flora	×			×	

All objectives will be compared between formulated food and placebo. ALT = alanine aminotransferase, AST = aspartate aminotransferase, BMI = body mass index, CK18 = cytokeratin 18, Cr = creatinine, eGFR = estimated glomerular filtration rate, ECG = Electrocardiogram, FINS = fasting insulin, FPG = fasting plasma glucose, GGT = γ-glutamyl transferase, HbA1c = glycosylated hemoglobin A1c, HCG = human chorionic gonadotropin, HDL-C = high-density lipoprotein-cholesterol, HOMA-IR = homeostasis model assessment insulin resistance, HRQOL = health-related quality of life, IL-6 = interleukin-6, LDL-C = low-density lipoprotein-cholesterol, PDFF = proton density fat fraction, TC = total cholesterol, TG = triglycerides, TSH = thyroid-stimulating hormone, WHR = waist to hip ratio.

### 2.5 Dosing rationale

In the pre-test, patients took 40 g formulated food every day for 12 weeks without any adverse drug reactions, including diarrhea, nausea, constipation, and so on. Therefore, this test selected dose of formulated food 40 g/day to avoid adverse reactions.

### 2.6 Randomization, masking and adverse events

Subjects should be screened before signing informed consent to determine if they meet the inclusion criteria and do not meet any exclusion criteria. Subjects will be randomly assigned to receive formulated food or placebo, and the distribution of the drug will be kept confidential to the investigator and subject. The drug manager will assign a drug or placebo to each patient during the follow-up period. All test drugs are packaged identically. Until the end of the trial, all subjects and investigators were blinded to the specific allocation of the drug. Adverse events (AEs) will be closely monitored throughout the course of the study and all AEs will be recorded in the case report form. If a serious AE occurs and the study drug is suspected to be a potential cause, the drug manager will only open the assignment information to the doctor in attendance.

### 2.7 Supply of formulated food and placebo

Only the person responsible for registering the patient knows the distribution of the drug and ensures that the subject is double-blind with the investigator. Formulated food and placebo are indistinguishable in appearance. They are manufactured and supplied by Jintong Special Medical Food Co., Ltd. (Guangzhou, China) and are not commercially available. We generated 120 nonrepeating random numbers through SPSS, and randomly assigned them to the formulated food group and the placebo group. Each number corresponding to a serial number. Subjects get unique serial number according to the time of enrollment that determines which group he or she is. Double-blind will remain throughout the study and all study data will be kept confidential until all subjects complete the 36-week study.

### 2.8 Efficacy and safety evaluation

#### 2.8.1 Primary endpoints

The study endpoints are summarized in Table 4. The primary endpoint is a decrease in MRS-PDFF by more than 30% from baseline at 24 weeks. MRS-PDFF and MRI-PDFF are closely related to liver fat content assessed by liver biopsy, with no superiority between them. Preliminary results from a phase II clinical trial confirmed the feasibility of MRI-PDFF in longitudinal observation of clinical trials and highlighted the evidence that liver stiffness by MRI-PDFF is a biomarker of steatosis. In addition, data confirm that changes in MRI-PDFF are associated with hepatic steatosis and overall NAS score response. At the same time, MRI achieves 100% accuracy by a fat fraction threshold of 5.56% to distinguish normal and abnormal fat fractions in liver steatosis testing as long as all known confounding factors are resolved. Therefore, MRS-PDFF and MRI-PDFF potentially replace liver biopsy to assess liver fat content.^[26–28]^

**Table 4:** Study endpoints.

Primary endpoints
<Efficacy endpoints>
Change in liver fat content (MRS/MRI-PDFF) from baseline at 24 wks
Secondary endpoints
<Efficacy endpoints>
Change in anthropological indicators (BMI, WHR) from baseline at 4,12,24 wks and at the time of drug discontinuation
Change in liver function biomarkers (ALT, AST, GGT) from baseline at 4,12, 24 weeks and at the time of drug discontinuation
Change in diabetic biomarkers (FPG, FINS, HOMA-IR, HbA1c) from baseline at 4,12, 24 wks and at the time of drug discontinuation
Change in lipid biomarkers (TG, TC, HDL-c, LDL-C) from baseline at 4,12, 24 wks and at the time of drug discontinuation
Change in IL-6 and CK18 from baseline at 4,12, 24 wks and at the time of drug discontinuation
Change in HRQOL using SF-36 from baseline at 24 wks
< Safety endpoints>
Change in blood tests of renal function (BUN, Cr, eGFR) from baseline at 4,12,24 wks
< Exploratory endpoint>
Changes of intestinal flora abundance and diversity from baseline at 24 wks

ALP = alkaline phosphatase, ALT = alanine aminotransferase, AST = aspartate aminotransferase: BMI = body mass index, CK18,cytokeratin 18, Cr = creatinine, eGFR = estimated glomerular filtration rate, FINS = fasting insulin, FPG = fasting plasma glucose, GGT = γ-glutamyl transferase, HbA1c = glycosylated hemoglobin A1c, HDL-C = high-density lipoprotein-cholesterol, HOMA-IR = homeostasis model assessment insulin resistance, HRQOL = health-related quality of life, IL-6 = interleukin-6, LDL-C = low-density lipoprotein-cholesterol, PDFF = proton density fat fraction, TC = total cholesterol, TG = triglycerides, WHR = waist to hip ratio. Placebo vs formulated food.

#### 2.8.2 Secondary endpoints

*Anthropometrics:* This mainly includes body mass index (BMI) and waist-to-hip ratio (WHR). At each visit, body weight and waist and hip circumferences will be measured using a standardized stadiometer and a calibrated electronic scale.

*Serum markers:* Alanine aminotransferase (ALT), aspartate aminotransferase (AST), γ-glutamyl transferase (GGT), fasting insulin, glucose, markers of inflammation (IL-6, CK18), lipid profile (total cholesterol (TC), low-density lipoprotein (LDL), high-density lipoprotein (HDL), and triglyceride (TG) will be assayed. Insulin resistance (HOMA-IR) was calculated using measurements for fasting glucose and fasting insulin. Hepatocyte apoptosis is a major mechanism of NAFLD progression, and cytokeratin-(CK-)18 is a marker derived from hepatocyte apoptosis produced by caspase3, which is significantly higher in patients with NAFLD than those without NAFLD.^[29,30]^ Importantly, in 2 large randomized controlled trials of children and adults, serum CK18 levels were significantly reduced with improved liver histology after treatment.^[31]^ These findings suggest that serum CK18 fragments may be an attractive biomarker for monitoring response to different therapeutic agents.^[32]^

*Quality of Life:* We will use SF-36 Life Scale to assess subjects.

#### 2.8.3 Safety endpoints

The safety of formulated food will be assessed on the basis of AE, clinical laboratory tests, physical examination, and vital signs. Clinical laboratory tests include liver, kidney, urine routine, glucose metabolism, lipid metabolism, electrocardiogram (ECG), and the like.

#### 2.8.4 Exploratory end point

*Stool samples:* Changes of intestinal flora abundance and diversity at 24 weeks.

### 2.9 Data

All the data will be entered by 2 persons and managed by the hospital's electronic data collection system. They will be kept confidential and only relevant personnel can view them. The data regulator will check them. They are independent of the sponsor and have no conflict of interest. The final data can be contacted with the sponsor after the trial is over.

### 2.10 Sample size estimation

The sample size was calculated using Medcalc version 18.2.1 software. On the basis of the results of the pre-test, we expected the formulated food to improve the ratio by about 50% and about 13% in the placebo group, with an α level of 2-sided type I error of 0.05, with a β level of type II error of 0.02. The placebo group was calculated to be 35 patients. Because the formulated food group vs the placebo group is 2:1, the total number is about 120 taking into account the 10% shedding rate.

### 2.11 Statistical analyses

This study will use intent-to-treat patient population. Values with a bias distribution will be log transformed before analysis. Baseline characteristics, categorical variables will be tested by χ^2^, and continuous variables will be compared by *t* test. Repeated measures analysis of variance (ANOVA) will be used for variables measured over time to test treatment efficacy, time, and interaction between them. To compare differences between baseline and 24 weeks, paired *t* tests will be used for primary and secondary outcomes (liver function biomarkers, diabetic biomarkers, liver fat content, diabetic biomarkers, lipid biomarkers, BMI, WHR). Secondary analysis will be performed in all participants who completed 24 weeks of intervention and had a drug intake rate of ≥80%, and cases with missing data would be excluded from the analysis. In all analyses, a 2-sided *P* value 0.05 will be considered significant. The frequency of the AE will be assessed using a χ^2^ test.

## 3 Discussion

The dietary supplements have received a lot of attention in recent years. Although some studies have shown that they can improve metabolism, they have not been formally used in clinical practice. The purpose of the double-blind, randomized, placebo-controlled clinical trial is to investigate the efficacy and safety of formulated food in patients with NAFLD. A follow-up plan was made to sequence the stool samples to explore the relationship between intestinal flora, NAFLD, and formulated food. We acknowledge some of the limitations of this research. Inclusion and exclusion criteria are proposed to maximize the safety of enrolled patients, while also reducing the likelihood of extending the results to patients with more severe conditions or those with a higher degree of comorbidity. If formulated food can achieve the desired effect, it will provide a new treatment idea for NAFLD.

## Acknowledgments

The authors thank those who collected data or performed the measurement: Qianru Zhu and Jin Chen.

## Author contributions

Junping Shi is responsible for conceiving and designing the trial, making the final decision to terminate the trial, and approving the final manuscript. Lili zhuo and Jiali Xu are drafting the manuscript and in charge of recruitment. Ningning You and Liyan Wang will participate in data collection and analysis. Yu Song and Yan Luo are responsible for supervising the study.

**Investigation:** Junping Shi.

**Methodology:** Ningning You.

**Software:** Liyan Wang.

**Supervision:** Yu Song, Yan Luo.

**Writing – original draft:** Lili Zhuo, Jiali Xu.

**Writing – review & editing:** Lili Zhuo, Jiali Xu.
